# Quantitative single molecule analysis of podoplanin clustering in fibroblastic reticular cells uncovers CD44 function

**DOI:** 10.1098/rsob.220377

**Published:** 2023-05-10

**Authors:** Shu En Lim, Megan D. Joseph, Charlotte M. de Winde, Sophie E. Acton, Sabrina Simoncelli

**Affiliations:** ^1^ Stromal Immunology Group, MRC Laboratory for Molecular Cell Biology, University College London, Gower Street, London WC1E 6BT, UK; ^2^ London Centre for Nanotechnology, University College London, London WC1H 0AH, UK; ^3^ Department of Chemistry, University College London, London WC1H 0AJ, UK; ^4^ Department of Molecular Cell Biology & Immunology, Amsterdam UMC location Vrije Universiteit Amsterdam, De Boelelaan 1117, Amsterdam, The Netherlands; ^5^ Cancer Center Amsterdam, Cancer Biology & Immunology, Amsterdam, The Netherlands; ^6^ Amsterdam Institute for Infection & Immunity, Cancer Immunology, Amsterdam, The Netherlands

**Keywords:** super-resolution, DNA-PAINT, qPAINT, podoplanin, CLEC-2, CD44

## Abstract

Upon initial immune challenge, dendritic cells (DCs) migrate to lymph nodes and interact with fibroblastic reticular cells (FRCs) via C-type lectin-like receptor 2 (CLEC-2). CLEC-2 binds to the membrane glycoprotein podoplanin (PDPN) on FRCs, inhibiting actomyosin contractility through the FRC network and permitting lymph node expansion. The hyaluronic acid receptor CD44 is known to be required for FRCs to respond to DCs but the mechanism of action is not fully elucidated. Here, we use DNA-PAINT, a quantitative single molecule super-resolution technique, to visualize and quantify how PDPN clustering is regulated in the plasma membrane of FRCs. Our results indicate that CLEC-2 interaction leads to the formation of large PDPN clusters (i.e. more than 12 proteins per cluster) in a CD44-dependent manner. These results suggest that CD44 expression is required to stabilize large pools of PDPN at the membrane of FRCs upon CLEC-2 interaction, revealing the molecular mechanism through which CD44 facilitates cellular crosstalk between FRCs and DCs.

## Graphical abstract

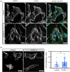

## 1. Introduction

Lymph nodes are highly organized tissues that contain and compartmentalize immune cell types to orchestrate adaptive immune responses. Lymph node tissue architecture is determined by stromal cell structures; fibroblastic reticular cells (FRCs) that establish cellular networks linking lymph and blood vasculature which provide trafficking routes for lymphocytes and myeloid cells and generate growth factor and survival factors for the immune cell populations they support [1]. Further, the fibroblastic reticular network is the key mechanically sensitive component of the lymph node capable of determining the physical properties of the tissue in steady state and adapting to permit lymph node expansion. Podoplanin (PDPN) has been determined as a mechanical sensor in FRCs, and mice with conditional genetic deletion of PDPN in fibroblastic stroma, *Pdgfra*^Δ*Pdpn*^ mice, exhibit attenuated lymph node expansion and altered immune activation [2–4]. PDPN overexpression has also been noted in inflammatory diseases, tissue damage and a wide range of cancers, and is directly correlated with disease outcomes, but the downstream signalling pathways and mechanisms of action of PDPN are still not fully understood. PDPN overexpression has been linked to cell migration, cell adhesion and cytoskeletal contractility in cancer cells [5–7]. For example, in non-motile tissue structures, such as FRCs in lymph nodes and on lymphatic endothelial cells, PDPN can act as a ligand to promote the migration of dendritic cells along stromal cell scaffolds through the direct binding of the C-type lectin-like receptor CLEC-2 [8]. PDPN can also interact with platelets through CLEC-2 which is a required interaction for the physiological separation of blood and lymphatic vasculature during development [9]. The same interaction with platelets also plays an important role in the function of high endothelial venues (HEVs) in lymph nodes, acting to prevent blood from leaking into the tissues [10].

It is known that PDPN has only a very short cytoplasmic tail of just 10 amino acids [11]. Therefore, it has been difficult to determine how PDPN is required for so many diverse functions in such a range of cell types and tissue contexts. It has been reported that PDPN can directly bind to ERM proteins (ezrin, radixin and moesin) to regulate RhoA GTPase and actomyosin contractility [2,5]. The transmembrane domain of PDPN may in fact be a key regulator of PDPN function, allowing PDPN molecules to rearrange within different regions of the plasma membrane and to permit the interactions between PDPN and other membrane binding partners [12]. We have recently reported that CD44, a non-kinase transmembrane glycoprotein and receptor for hyaluronic acid, is a key PDPN binding partner required for the response of FRCs to CLEC-2^+^ dendritic cells [13]. PDPN and CD44 interactions are mediated through their transmembrane domains and are also dependent on cholesterol levels in the plasma membrane [2,14]. Interestingly, *Pdpn* and *Cd44* mRNA expression are also coregulated, and knockdown of *Cd44* results in lower expression levels of PDPN [13]. Furthermore, overexpression of CD44 can attenuate PDPN-driven actomyosin contractility [2,13]. CD44 and PDPN colocalize at the plasma membrane of FRCs and their interaction is increased when PDPN binds to CLEC-2, leading us to hypothesize that CD44 controls PDPN activity through the spatial organization and clustering of PDPN molecules within the membrane. However, we have lacked the technical capability to quantify PDPN clustering at a molecular scale to formally test this hypothesis.

Here we use a quantitative single-molecule localization microscopy (SMLM) technique, known as DNA-PAINT (point accumulation for imaging in nanoscale topography) [15], to study how the spatial organization of PDPN proteins in the plasma membrane of wild-type (WT) and CD44 knock-out (CD44 KO) FRCs responds to binding of CLEC-2. DNA-PAINT relies on the binding and unbinding of two types of short single-stranded DNA sequences, one chemically coupled to the antibody targeting the protein of interest, known as the ‘docking’ strand, and another one fluorescently labelled and freely diffusing in solution, known as the ‘imager’ strand. The transient, yet repetitive binding between imager and docking DNA strands creates the characteristic blinking effect needed for super resolution fluorescence microscopy, as the fluorescent signal is preferentially detected during binding events. The continuous replenishment of imager strands makes DNA-PAINT immune to photobleaching, thus the same target protein can be detected multiple times with virtually unlimited number of photons outperforming the localization precision obtained with more conventional SMLM methods such as PALM (photoactivated localization microscopy) [16] and STORM (stochastic optical reconstruction microscopy) [17]. The high level of nanometre accuracy attained by DNA-PAINT (i.e. it can routinely obtain sub-10 nm localization precision) has proven capable of imaging individual molecular targets on synthetic samples that simulates biomolecular nanoclusters [18], as well as protein clusters in fixed cells [19–21]. Furthermore, due to the predictable binding kinetics between imager and docking strands, it is possible to correlate the frequency of single-molecule events with the underlying number of labelled molecular targets [22] overcoming ‘overcounting’ artefacts observed with other SMLM techniques. During the last years, examples of the use of DNA-PAINT to quantify protein clustering in different biological systems started to emerge [19] and here we use it to shed light into the role of CLEC-2 and CD44 on PDPN clustering.

## 2. Results

### 2.1. CLEC-2 drives PDPN and CD44 co-localization in FRCs and cell spreading

**Figure 1.**
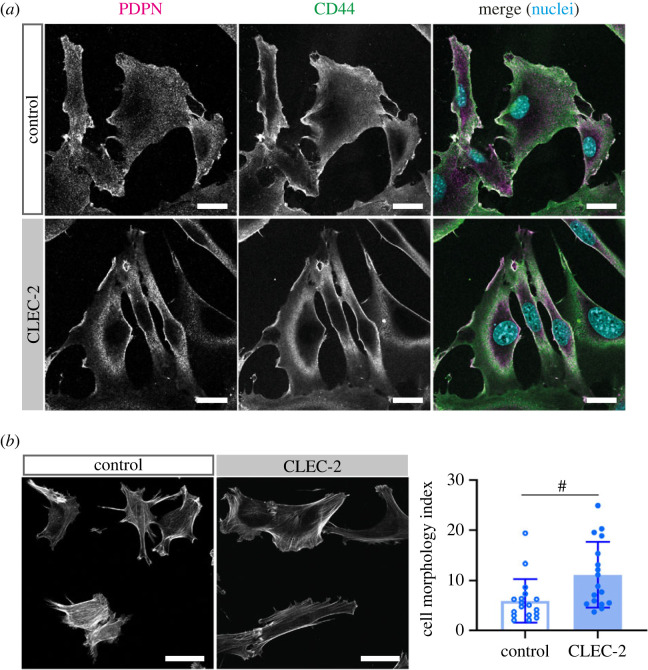
CLEC-2 increases PDPN-CD44 co-localization and FRC elongation. (*a*) Double immunofluorescence staining of PDPN (magenta) and CD44 (green) in control or CLEC-2-expressing (CLEC-2) FRC cell lines. Maximum Z stack projections of representative images are shown. The scale bars represent 25 µm. (*b*) Left panel: immunofluorescence staining of F-actin in FRCs cultured on uncoated (control) or CLEC-2-Fc coated (CLEC-2) glass slides for 4 h. The scale bars represent 50 µm. Right panel: cell morphology index of unstimulated (control; open) and CLEC-2 stimulated (closed) FRCs. Data shown as mean ± s.d. of *n* = 17–18 individual FRCs per condition. Mann–Whitney test, two-tailed, ^#^*p* *<* 0.0001.

Confocal imaging of FRCs in steady state confirms that PDPN and CD44 colocalize with each other on FRC membranes (figure 1*a*), as previously published [13]. We studied PDPN-CD44 co-localization in a CLEC-2-Fc expressing FRC cell line [23], modelling prolonged CLEC-2 stimulation to mimic a crosstalk between dendritic cells and fibroblastic stroma during an adaptive immune response *in vivo*. We observed increased changes in the co-localization of PDPN-CD44 on FRC membranes (figure 1*a*), which is in line with our previous observations that PDPN-CD44 co-localization is significantly increased upon CLEC-2 stimulation [13]. However, due to the diffraction-limited resolution of confocal imaging, it is not possible to accurately uncover the interaction between PDPN and CD44. To improve our understanding of the molecular interactions between PDPN and CD44, and the effect of CLEC-2 stimulation, we here investigated PDPN clustering with single-molecule localization microscopy (SMLM) under total internal reflection fluorescence (TIRF) excitation in WT and CD44 KO FRC cell lines. TIRF illumination limits the excitation of fluorophores to a very thin optical section of approximately 150 nm above the glass/specimen interface which is ideal for imaging PDPN on cell membranes. Accordingly, FRCs were stimulated with CLEC-2-Fc or control coated glass slides. To assess that this method of CLEC-2 stimulation was functional, we quantified the cell morphology index of unstimulated and stimulated FRCs. Previous work has shown that there is an increase in the morphology index between FRCs co-cultured with and without CLEC-2^+^ DCs [13] because of inhibition of FRC contractility, as measured by reduced F-actin^+^ stress fibres allowing cell spreading [13]. Our results showed a similar increase in the morphology index when FRCs were cultured on CLEC-2 coated glass slides (figure 1*b*) compared to CLEC-2 expressing FRCs or DC interaction [13] which suggests that immobilized CLEC-2 on coated glass slides induces a CLEC-2 mediated response in FRCs. As such, we used this method to study in detail PDPN-CD44 interactions on the cell membrane of FRCs in absence or presence of CLEC-2-Fc.

### 2.2. Super-resolution DNA-PAINT imaging of PDPN

**Figure 2.**
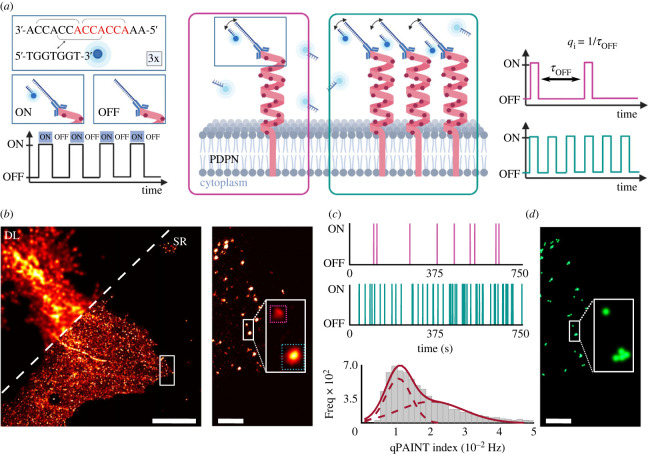
qPAINT imaging and calibration to acquire quantitative high resolution PDPN protein maps. (*a*) Schematic representation of DNA docking and imager strand sequences displaying the 3× repeat binding motif targeting PDPN proteins with ON-OFF time series for a single binding site and three binding sites, respectively. (*b*) Example diffraction limited (top left, DL) and super resolution (bottom right, SR) image of PDPN on an FRC. Box represents highlighted region for zoom-in visualization. Scale bar represents 6 *μ*m for full size image and 500 nm for zoom-in region. (*c*) ON-OFF time series for clusters of single molecule localizations shown in zoom-in region in (*b*) (top and middle). Histogram of qPAINT calibration indexes per cluster pooled from all super resolution images fit with a multi-peak Gaussian function. (*d*) Quantitative PDPN protein map of zoom-in region indicated in (*b*) highlighting clusters shown in (*c*). Scale bar represents 500 nm.

DNA-PAINT super resolution imaging under TIRF was used to elucidate the impact of CLEC-2 on the spatial organization of PDPN proteins in WT and CD44 KO FRCs. For PDPN image acquisition, FRCs were labelled with anti-PDPN antibody chemically coupled to a docking DNA sequence containing a repetitive binding sequence (figure 2*a*, left). This repetitive sequence (ACCACCA) increases the frequency of binding events as imagers have 3 times the possibility to bind with respect to a non-repetitive one. Ultimately, this leads to faster image acquisition while maintaining low imager concentrations, resulting in high signal-to-noise ratio and single molecule localization precision [24]. Using a 1 nM imager DNA sequence solution (TGGTGGT fluorescently labelled with ATTO 643) an overall localization precision of 9 nm was achieved, based on nearest neighbour analysis (electronic supplementary material, figure S1). Figure 2*b* (left) displays an example super resolution image of PDPN proteins in WT FRCs in comparison to the diffraction limited visualization.

### 2.3. Quantification of PDPN proteins via qPAINT analysis

To quantitatively characterize the influence of CLEC-2 – PDPN interaction on the spatial organization of PDPN, we determined how many individual PDPN proteins reside within a cluster of single-molecule localizations detected during DNA-PAINT imaging via qPAINT analysis. In DNA-PAINT imaging, the repetitive binding of multiple imager strands to a docking sequence, creates a cluster of single molecule localizations around the true position of the protein. qPAINT is based on the analysis of the binding kinetics between imager and docking strands, particularly via the determination of the average dark time (i.e. waiting time between binding events), of a cluster of single molecule localizations. $\tau_{\mathrm{OFF}}$ is inversible proportional to the number of proteins, *N*, within that cluster of single molecule localization, according to

2.1 $$N={\left(k_{\mathrm{ON}}\cdot [I]\cdot \tau_{\mathrm{OFF}}\;\right)}^{-1}=\frac{\tau_{\mathrm{OFF},1\;}}{\tau_{\mathrm{OFF}}}$$

where $k_{\mathrm{ON}}$ denotes the binding rate of imager to docking strand, [I] is the concentration of the imager strand and $\tau_{\mathrm{OFF},1\;}$ is the dark time for the case of a single protein and is equal to ${\left(k_{\mathrm{ON}}\cdot [I]\right)}^{-1}$. Equation (2.1) implies that every additional protein within the cluster leads to a proportionally shorter $\tau_{\mathrm{OFF}}$, as represented in figure 2*a*. To quantify PDPN proteins within each cluster of single molecule localizations detected via DNA-PAINT imaging, we used a recently developed automated qPAINT pipeline [25].

Firstly, the clustering algorithm DBSCAN (density-based spatial clustering of application with noise) was used to isolate clusters of single molecule localizations in 3.5 × 3.5 µm regions of interest (ROIs) randomly selected from DNA-PAINT images, and for each of the detected clusters its average dark time was calculated. To allow for direct proportionality, the inverse of the measured dark times was used, a term known as the influx rate [22] (*ξ*) or the qPAINT index *q_i_* [19]. As an example, figure 2*b* shows two highlighted single molecule localization clusters with their corresponding ON/OFF time series depicted in figure 2*c* from which their *q_i_* can be obtained. To determine the qPAINT index of a cluster of single molecule localizations containing only one PDPN protein, *q_i1_* (i.e. ${\left(\tau_{\mathrm{OFF},1}\right)}^{-1}$), we calculated the histogram of qPAINT indexes of small single-molecule clusters (a maximum distance of less than 100 nm) obtained from DNA-PAINT imaging of PDPNs in FRC's (figure 2*c*). Multi-peak Gaussian function fitting of the histogram renders peaks at multiples of the qPAINT index 0.012 Hz, corresponding to the *q_i1_.* The ratio between *q_i1_* and the qPAINT index of a particular cluster (*q_i1_*/*q_i_*) defines the number of proteins within that cluster of single molecule localizations. From the multi-peak Gaussian fit parameters (i.e. standard deviation of the Gaussian distribution) we estimate a counting precision of 71%, meaning that our pipeline can quantify one protein with a precision of ±0.3 proteins. Notably, DNA-PAINT is immune to photobleaching as the continuous replenishment of dye-labelled imager strands from solution provides a constant influx rate of imager probes (electronic supplementary material, figure S2). As such, it is possible to image for an arbitrarily long time to further increase the counting precision of protein copy numbers [22].

Finally, to reconstruct nanoscale quantitative maps of PDPN proteins, each cluster of single molecule localizations was partitioned into *N* clusters with *N* corresponding to the number of proteins per cluster, using a clustering algorithm known as *k*-means. Figure 2*d* shows the reconstructed quantitative protein map from the ROI of single molecule localizations rendered in figure 2*b*. Overall, this pipeline allows the quantification of the total number of PDPN proteins and PDPN clusters per unit area, as well as other parameters associated with the cluster composition (proteins per cluster) and size (equivalent diameter).

### 2.4. PDPN is enriched at the plasma membrane upon CLEC-2 stimulation

**Figure 3.**
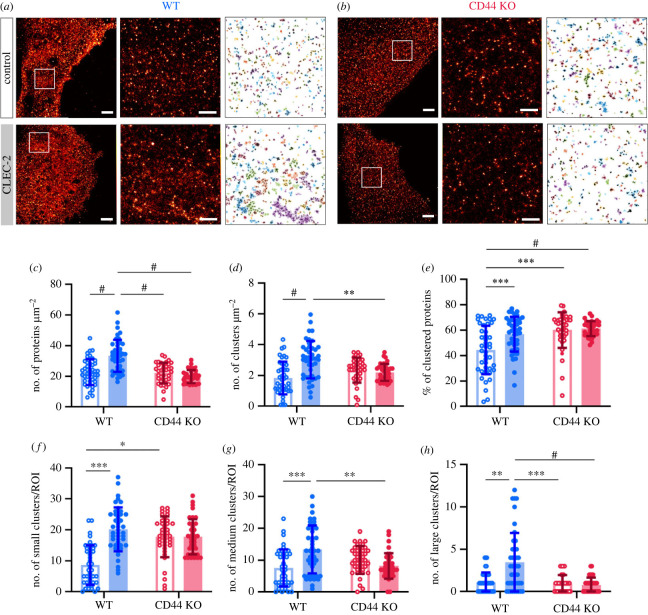
Comparison of PDPN proteins in WT and CD44 KO FRCs under control and CLEC-2 stimulation. (*a,b*) Representative DNA-PAINT images of PDPN proteins in WT (*a*, blue) and CD44 KO (*b*, red) under control conditions (unstimulated; first row) and upon CLEC-2 stimulation (second row) with example ROI of 3.5 × 3.5 µm shown in white box displayed in the middle and subsequent DBSCAN cluster analysis of this ROI on the right. Scale bar represents 2 µm for left-hand side image and 0.5 µm for middle image. (*c*) Density of PDPN proteins. (*d*) Density of PDPN protein clusters. (*e*) Percentage of clustered PDPN proteins. (*f–h*) Number of small (less than 6 proteins, *f*), medium (6–12 proteins, *g*) and large (greater than 12 proteins, *h*) PDPN clusters per ROI. WT FRCs are represented in blue, CD44 KO FRCs in red with control conditions represented with unfilled circle points and bar and CLEC-2 stimulated conditions with filled circle points and bar. Significance values from two-way ANOVA for normally distributed data and Kruskal–Wallis tests for non-normally distributed data adjusted *p*-values, *p ≤ 0.05, **p ≤ 0.01, ***p ≤ 0.001, ^#^*p* < 0.0001; all other *p*-values non-significant, *n* = 43, 43, 38 and 38 ROIs, respectively.

Next, we used DNA-PAINT imaging of PDPN in WT FRCs to investigate the effect of CLEC-2 stimulation on PDPN clustering (figure 3*a*, left). To statistically quantify the spatial organization of PDPN, we randomly selected forty ROIs from ten WT FRCs per condition and performed qPAINT analysis as described above. Figure 3*a* displays an example DNA-PAINT image of PDPN (left) in WT FRCs in steady state (top) and stimulated (bottom) conditions, together with the DNA-PAINT zoom-in (centre) of selected ROIs and corresponding cluster analysis and protein quantification (right). In steady state condition, we quantified that the basal level of PDPN in WT FRCs is 23 ± 8 proteins µm^−2^ with almost half (44 ± 19%) of these proteins forming clusters (minimum size of cluster defined as three proteins; figure 3*e*) of a median cluster diameter of 172 ± 93 nm (electronic supplementary material, figure S3) and a median first neighbour distance of 32 nm (electronic supplementary material, figure S4).

Stimulation with CLEC-2, on the other hand, resulted in an increase of total number of PDPN proteins and clusters in WT FRCs (from 23 ± 8 to 33 ± 10 proteins µm^−2^ and from 2 ± 1 to 3 ± 1 clusters µm^−2^, respectively; figure 3*c,d*). However, there is only a 13% increase (from 44 ± 19 to 57 ± 14%) on the overall percentage of clustered PDPN proteins upon CLEC-2 stimulation in WT FRCs (figure 3*e*), which suggests that the increase in the number of PDPN clusters (figure 3*d*) is mainly related to a higher density of PDPN proteins within the interaction area. Regarding the composition of PDPN clusters, we defined three cluster types: small (<6 proteins), medium (6–12 proteins) and large (>12 proteins) based on the distributions of PDPN proteins per cluster (electronic supplementary material, figure S5). After CLEC-2 stimulation, a significant increase (approx. 2–3-fold) in the number of all cluster's types (i.e. small, medium, and large) in WT FRCs was quantified (figure 3*f–h*).

### 2.5. CD44 is required to stabilize medium and large PDPN clusters upon CLEC-2 stimulation

Because CD44 is required for FRCs to respond to CLEC-2^+^ DCs [13], we next characterized the effect of CD44 deletion on PDPN clustering upon CLEC-2 interaction using the same analysis pipeline as above. Example DNA-PAINT images and cluster analysis and quantification of PDPN in CD44 KO FRCs in steady state and CLEC-2 stimulating conditions are presented in figure 3*b*. In the case of CD44 KO FRCs stimulation with CLEC-2 resulted in no significant differences in either the total number of PDPN proteins (from 20 ± 4 to 22 ± 7 proteins µm^−2^), nor the number of clusters (2 ± 1 clusters µm^−2^, in both cases) or the percentage of clustered PDPNs (from 60 ± 14 to 61 ± 6%) in comparison to steady state conditions. The total number of PDPN proteins in CD44 KO FRCs are comparable with the results observed for WT FRCs in steady state conditions (figure 3*c*). However, in steady state, PDPN clustering increases from 44 ± 19% in WT FRCs to approximately 60% in CD44 KO cells (figure 3*e*).

With respect to the number of PDPN proteins per cluster in CD44 KO FRCs, CLEC-2 treatment made no significant difference. On the contrary, in steady state conditions, we found that absence of CD44 leads to an increase of small PDPN clusters (with <6 PDPN proteins) compared to WT FRCs (figure 3*f*), while the number of medium (6–12 PDPN proteins) and large protein clusters (>12 PDPN proteins) per unit area remain unaffected with CD44 deletion (figure 3*g,h*). On the other hand, when comparing WT and CD44 KO FRCs upon CLEC-2 stimulation, CLEC-2 stimulation favours the formation of medium (*p*-value = 0.0069) to large protein clusters (*p*-value < 0.0001) only for the case of WT FRCs (figure 3*g,h*) with the number of small PDPN clusters (<6 PDPN proteins) remaining the same (figure 3*f*; *p* value = 0.3505). Together, these data show that CLEC-2 promotes clustering of PDPN on the FRC plasma membrane in a CD44-dependent manner and suggest that CD44 is required to stabilize larger PDPN clusters.

## 3. Conclusion

A quantitative single molecule-based super-resolution imaging technique based on DNA-PAINT, i.e. qPAINT, was used as a novel method for the quantitative analysis of the spatial organization of PDPN in FRCs. In accordance with previous studies [2,8,13,23,26], we detected an increase in the total number of PDPN proteins in FRC membranes under CLEC-2 stimulation and for the first time precisely quantified and localized the number of PDPN proteins in the plasma membrane.

We have previously reported that CLEC-2 stimulation of FRCs can increase PDPN expression at both mRNA and protein levels through regulation of transcription [4,13]. However, the timeframe for experiments presented here is insufficient for increased transcription to account for the higher number of PDPN molecules on the plasma membrane that we observe. Another possible pathway to increase PDPN density is the recruitment from cytoplasmic stores, but we have no evidence for this type of mechanism. Alternatively, we suggest that the increased PDPN density could be due to existing membrane bound molecules redistributed to the area of CLEC-2 stimulation.

Interestingly, we determine that almost half of the total number of PDPN proteins are pre-clustered in the plasma membrane of FRCs in steady state conditions. It remains to be determined whether this steady state clustering is required for PDPN signalling in the absence of stimulation [4]. Stimulation with CLEC-2 promotes the formation of all cluster sizes, with the most significant change in the large PDPN clusters composed of more than 12 PDPN proteins. This has been predicted from observations of confocal microscopy datasets, but, via qPAINT imaging, we have now been able to formally visualize and quantify the response with single protein resolution. It should be noted though, that as any technique that relies on antibody labelling, the quantification toward absolute number of protein copy numbers via qPAINT analysis will be reliant on antibody-target binding efficiency, leading to potential undercounting bias. Still, by keeping the imaging and data processing conditions the same in all experiments, it is possible to compare and draw conclusions about changes in the spatial organization of PDPN under the different conditions.

Our findings place CD44 as a key player to stabilize the formation of large PDPN clusters with more than 12 proteins upon CLEC-2 stimulation. In the absence of CD44, PDPN proteins fail to cluster appropriately when FRCs bind CLEC-2, impacting signalling downstream of CLEC-2/PDPN binding. These data provide a molecular level understanding to explain why CD44 is required for FRCs to respond to CLEC-2^+^ dendritic cells [13], highlighting an additional mechanism of activation for PDPN-dependent signalling. Further, investigating molecular clustering as a mechanism may help address how PDPN can be expressed in so many different cell types and linked to so many varied physiological and pathological phenotypes [27–29].

## 4. Material and methods

### 4.1. Cell culture

Control [2], CLEC-2-Fc expressing [23]and CD44 KO FRC [13] cell lines are previously described. Cell lines were cultured in high-glucose GlutaMAX DMEM (Gibco, via ThermoFisher Scientific) supplemented with 10% FCS (Sigma-Aldrich), 1% penicillin-streptomycin and 1% insulin-transferrin-selenium (Gibco, via Thermo Fisher Scientific) at 37°C, 10% CO_2_ and passaged using cell-dissociation buffer (Gibco, via Thermo Fisher Scientific).

### 4.2. CLEC-2 stimulation

Generation of CLEC-2-Fc was performed as previously described [2,8]. Control and CLEC-2-Fc supernatant was diluted in cell culture medium. For control and CLEC-2 coated glass slides, glass bottomed microscopy chambers (µ-SlideVI^0.5^, Ibidi, Fitchburg, WI, USA) were incubated with diluted control or CLEC-2 supernatant for 1 h at room temperature. The diluted supernatant was then exchanged with culture medium seeded with 10 000 cells per chamber for 4 h with control or CLEC-2 supernatant added to the culture medium.

### 4.3. DNA – antibody coupling reaction

DNA labelling of hamster anti-mouse PDPN monoclonal antibodies (DM3501, Origene, MD, USA) was performed using the malemidePEG2-succinimidyl ester coupling reaction [30]. Briefly, thiolated-DNA 5′-Thiol-AAACCACCACCACCA-3′ (Eurofins, Ebersberg, Germany) (13 µl, 1 mM) was reduced by incubation with freshly prepared DTT solution (30 µl, 250 mM) (Thermo Fisher Scientific, Waltham, MA, USA) for 2 h at room temperature on a shaker in the dark. 30 min after starting this reduction step maleimide-PEG2-succinimidyl ester crosslinker solution (0.85 µl, 23.5 mM) (Sigma-Aldrich, St. Louis, MO, USA) was incubated with monoclonal antibody solution (50 µl, 26 µM) for 90 min at 4°C on a shaker in the dark. Excess DTT and crosslinker were removed using spin filtration by Microspin Illustra G-25 columns (GE Healthcare, Chicago, IL, USA) and Zeba spin desalting columns (7K MWCO, Thermo Fisher Scientific, Waltham, MA, USA), respectively. The resultant products were mixed and incubated overnight at 4°C on a shaker in the dark. Finally, excess DNA was removed via Amicon spin filtration (100K, Merck, Kenilworth, NJ, USA) and antibody–DNA concentration was measured using a NanoDrop One spectrophotometer (Thermo Fisher Scientific, Waltham, MA, USA). Quantification of the DNA-antibody coupling ratio via spectrophotometric analysis revealed a ratio of approximately 1 for the DNA anti-mouse PDPN monoclonal antibody.

### 4.4. Cell fixation and immunofluorescence staining for confocal imaging experiments

FRCs were seeded on glass coverslips for 24 h at 37°C, 10% CO_2_. Next, cells were fixed in 3.6% formaldehyde (Sigma-Aldrich; diluted in PBS), and subsequently blocked in 2% BSA in PBS and stained for 1 h at RT with the following primary mouse antibodies: hamster anti-podoplanin-eFluor660 (clone 8.1.1, 1 : 200, eBioscience, 50-5381-82) and rat anti-CD44 (clone IM7, 1 : 200, BD Biosciences, 553 131). This was followed by incubation with appropriate Alexa Fluor-conjugated secondary antibodies (1 : 500, Invitrogen, via Thermo Fisher Scientific) for 1 h at RT. F-actin and cell nuclei were visualized using respectively phalloidin-TRITC (P1951-1MG) and DAPI (D9542-1MG; both 1 : 500 dilution, both from Sigma-Aldrich) incubated for 15 min at RT, and coverslips were mounted in Mowiol (Sigma-Aldrich). Cells were imaged on a Leica SP5 confocal microscope using HCX PL APO 63× oil lens. Images were analysed using Fiji/ImageJ software. Z stacks (0.5 µm step^−1^) were projected with ImageJ Z Project (maximum projection).

### 4.5. Cell fixation and immunofluorescence staining for cell morphology confocal imaging experiments

Cells were fixed in 4% PFA and 0.1% glutaraldehyde for 30 mins at room temperature. Following 3 × washes in PBS, cell membranes were permeabilized for 5 min in 0.1% Triton X-100 solution (Avantor, Radnor Township, PA, USA), then washed again 3× in PBS. Samples were then blocked in 5% bovine serum albumin (Merck, Kenilworth, NJ, USA) for 30 min and subsequently incubated with phalloidin-Alexa Fluor 488 (Cell Signalling Technology, Denver, MA, US) for 30 min in the dark. Finally, cells were washed 3× with PBS before imaging.

### 4.6. Confocal imaging and quantification of cell morphology

Cells were imaged on a Leica TCS SP5 confocal microscope (DMI6000, Leica Microsystems, Wetzlar, Germany) with a 63× oil immersion objective (HCX PL APO, Leica Microsystems, Wetzlar, Germany) and analysed using ImageJ software. Z stacks of 120 µm (10 µm step^−1^) were projected with ImageJ Z Project (maximum projection), and the cell morphology index of individual cells were calculated as follows: perimeter^2^/4π × area. The area and perimeter of cells were measured by manually drawing around the cell shape using F-actin phalloidin staining.

### 4.7. Cell fixation and immunofluorescence staining for DNA-PAINT imaging experiments

Cells were fixed in 4% PFA and 0.1% glutaraldehyde for 30 min at room temperature. Following 3× washes in PBS, cell membranes were permeabilized for 5 min in 0.1% Triton X-100 solution, then washed again 3× in PBS. Autofluorescence was quenched using 50 mM ammonium chloride solution (Avantor, Radnor Township, PA, USA) for 5 min and washed 2× in PBS. Next, cells were blocked in 5% bovine serum albumin for 60 min and subsequently incubated with DNA-labelled anti-PDPN receptor antibody diluted in blocking buffer overnight at 4°C. The next day the cells were washed 3× in PBS. 150 nm gold nanoparticles (Sigma-Aldrich, St Louis, MO, USA) were added as fiducial markers for drift correction. After 3× washes in PBS, 1 nM imager strand solution in the presence of an oxygen scavenging and triplet state quencher system was added to the samples. This system consisted of 1 × PCA (Stock 40 × PCA solution), 1 × PCD (Stock 100 × PCD solution) and 1 × Trolox (Stock 100 × Trolox solution) in 1 × PBS + 500 mM NaCl buffer and incubated in the dark for 1 h before imaging.

40 × PCA (protocatechuic acid) stock was made from 154 mg of PCA (Sigma-Aldrich, St Louis, MO, USA) in 10 ml of distilled water adjusted to pH 9.0 with NaOH (Avantor, Radnor Township, PA, USA). 100x PCD (protocatechuate 3,4-dioxygenase) solution was made by adding 2.2 mg of PCD (Sigma-Aldrich, St Louis, MO, USA) to 3.4 ml of 50% glycerol (Sigma-Aldrich, St Louis, MO, USA) with 50 mM KCl (Sigma-Aldrich, St Louis, MO, USA), 1 mM EDTA (Invitrogen, Waltham, MA, USA) and 100 mM Tris buffer (Avantor, Radnor Township, PA, USA). 100× Trolox solution was made by dissolving 100 mg of Trolox (Sigma-Aldrich, St Louis, MO, USA) in 0.43 ml methanol (Sigma-Aldrich, St Louis, MO, USA), 0.345 ml 1 M NaOH and 3.2 ml of distilled water. The imager DNA (5′-TGGTGGT-3′) strand was conjugated to the fluorescent molecule Atto643 at the 3′ terminus (Eurofins, Ebersberg, Germany).

### 4.8. DNA-PAINT imaging experiments

FRCs were imaged on a custom built total internal reflection fluorescence (TIRF) microscope based on a Nikon Eclipse Ti-2 microscope (Nikon Instruments, Tokyo, Japan) equipped with a 100× oil immersion TIRF objective (Apo TIRF, NA 1.49) and a Perfect Focus System. Samples were imaged under TIRF illumination with a 647 nm laser (Coherent OBIS LX, 120 mW, Santa Clara, CA, USA), magnified with both a custom-built telescope (AC254-050-A-ML and AC508-075-A-ML, Thorlabs, Newton, NJ, USA) and a variable beam expander (BE02-05-A, Thorlabs, Newton, NJ, USA). Laser polarization was adjusted to circular using a polarizer (LPVISC050-MP2, Thorlabs, Newton, NJ, USA) followed by a quarter waveplate (LAS-043013, Laser 2000, Cambridge, UK). The beam was focused into the back focal plane of the microscope objective using a suitable lens (AC508-300-A-ML, Thorlabs, Newton, NJ, USA), passed through a clean-up filter (FF01-390/482/563/640-25, Semrock, Rochester, NY, USA) and coupled into the objective using a beam splitter (Di03-R405/488/561/635-t1-25×36, Semrock, Rochester, NY, USA). Fluorescence light was spectrally filtered with an emission filter (FF01-446/523/600/677-25, Semrock, Rochester, NY, USA) and imaged on a sCMOS camera (ORCA-Flash4.0 V3 Digital, Hamamatsu, Hamamatsu City, Japan) without further magnification, resulting in a final pixel size of 130 nm in the focal plane, after 2 × 2 binning. 15 000 frames were acquired with 100 ms integration time and a laser power density at the sample of 0.8 kW cm^−2^.

### 4.9. DNA-PAINT image reconstruction and cluster analysis

DNA-PAINT raw data was processed using the following pipeline in Picasso (v. 0.4.0) [30] and MATLAB (v. 9.10.0) [31]. First, the ‘Localize’ component of Picasso was used to identify and localize single molecule events from the raw fluorescent multi-tiff images. Subsequently, images were drift corrected using a stepwise protocol in Picasso's ‘Render’ involving an image sub-stack cross correlation analysis followed by use of gold nanoparticles as fiducial markers. Localizations with uncertainties greater than 13 nm were removed while no merging was performed for molecules re-appearing in subsequent frames. Finally, regions of interest (ROIs) of approximately 3.5 by 3.5 µm^2^ were selected within the ‘Render’ module of Picasso and analysed using DBSCAN from PALMsiever [32] in MATLAB. DBSCAN is a data clustering algorithm that detects clusters of localizations by looking for the minimum number of points (minPts) within a circle with radius epsilon (eps). For ‘eps’, we used the localization precision of our DNA-PAINT images as determined via the nearest-neighbour based analysis, which was approximately 10 nm for all the images (electronic supplementary material, figure S1). For ‘minPts’ we chose 15 localizations as this is a parameter in accordance with the binding kinetics of the imager–docking pair (*τ*_bright_ = 0.27 s; electronic supplementary material, figure S4; *k*_ON_ = 10^7^ M^−1^ s^−1^) [25], the number of recorded frames, the DNA imager concentration, and statistical considerations for the distribution of single-molecule localizations. In brief, the reversible binding kinetics between docking and imager strands in DNA-PAINT allows us to estimate the fraction of time (binding probability) that a DNA-docking strand will be bound to an imager strand during the acquisition time of the experiment. By considering the ligand–receptor binding kinetics model, and knowing the experimental concentration of the imager strand, [Imager], and the total number of detected frames, $N_{\mathrm{frames}}$, it is straightforward to predict that the number of single molecule localizations expected per docking strand, $N_{\mathrm{localizations}}^{1\;\mathrm{docking}\;\mathrm{strand}}$ is equal to $N_{\mathrm{localizations}}^{1\;\mathrm{docking}\;\mathrm{strand}}=\;N_{\mathrm{frames}}*\;\tau_{\mathrm{bright}}*\;\kappa_{\mathrm{ON}}[\mathrm{Imager}]$. In our case, this value corresponds to 40 localizations. To determine the cut-off value of the minimum number of localizations required to detect a true DNA docking strand (i.e. non-spurious binding), that renders on average 40 single molecule localizations, we assume that localizations are distributed as a Poisson process, and we calculate the largest value that gives a cumulative probability of 1 for detecting a cluster of points corresponding to a true docking site with more than that number of localizations, ‘minPts’. This means that clusters of points with fewer minPts are likely to correspond to noise, and therefore, should be discarded from the analysis. Furthermore, and as a second step to remove nonspecific signals of imager strands, we implemented a mean frame filtering step in our MATLAB data analysis pipeline. In short, repetitive transient binding of imagers to a DNA-labelled antibody leads to a mean frame of approximately half the number of total frames in the acquisition window (i.e. in our case 7500 frames). To remove single molecule localization clusters detected by DBSCAN that are not continuously visited by an imager strand during the whole course of the acquisition, we fitted the mean frame value of all detected clusters and set the cut-off value as the mean ± the standard deviation.

### 4.10. qPAINT analysis

We used a custom-written MATLAB code that analyses the fluorescence time series of each detected localization cluster to estimate the number of PDPN molecules for each cluster as described previously [25]. In short, time stamps (frame number) of localizations within the same cluster as defined by DBSCAN were used to reconstruct the sequence of dark times per cluster as continuous frame times that did not contain an event. All the dark times per cluster were pooled and used to obtain a normalized cumulative histogram of the dark times which was then fitted with the following exponential function: 1 – exp(*t*/*τ*_d_) to estimate the dark time, *τ*_d_, per cluster. The inverse of the dark time was calculated for each cluster and stored as the qPAINT index of the cluster (*q*_i_). An initial calibration step was performed in which the qPAINT index of clusters with a maximum point distance of 100 nm were plotted as a cumulative histogram and fitted with a multi-peak Gaussian. This displayed peaks at multiples of a qPAINT index of 0.012 Hz, which corresponds to the qPAINT index of a cluster of single molecule localizations representing one binding site i.e. PDPN monomers, *q*_i1_. This calibration value was used to calculate the number of PDPN proteins per cluster as the ratio of the qPAINT index of the cluster, *q*_i_ and *q*_i1._ To recover a likely distribution of PDPN receptors in each cluster of localizations, we used *k*-means clustering, where *k* corresponds to the protein copy number per cluster. All the code for qPAINT data analysis is available at https://github.com/Simoncelli-lab/qPAINT_pipeline.

### 4.11. Statistical analysis

For confocal imaging, 18 unstimulated and 17 stimulated FRCs were analysed. For DNA-PAINT imaging, a minimum of 38∼3.5 by 3.5 µm^2^ regions, obtained from 9–10 WT and CD44 KO FRCs were analysed per condition (CD44 WT and CD44 KO with and without CLEC-2 stimulation). Statistical analyses were performed using Prism 6.0 software (GraphPad). The distributions of data points and their variance were determined, and parametric or non-parametric tests were used as appropriate. Multiple groups were compared using two-way ANOVA with Tukey's multiple comparisons test for normally distributed data. For non-normally distributed data Kruskal–Wallis *H*-test with Dunn's multiple comparisons tests were performed.

## Data Availability

The data supporting the findings of this study are available within the article and its electronic supplementary material [33]. Source data are provided with this paper. All the code for qPAINT data analysis is available at https://github.com/Simoncelli-lab/qPAINT_pipeline.
